# Impact of traditional East Asian medicine as an add-on therapy on survival and recurrence after surgery for breast cancer: A systematic review and meta-analysis

**DOI:** 10.3389/fphar.2023.1125373

**Published:** 2023-04-04

**Authors:** Jee-Hyun Yoon, Eun Hye Kim, Su Bin Park, Hayun Jin, Seong Woo Yoon

**Affiliations:** Department of Korean Internal Medicine, Kyung Hee University Hospital at Gangdong, Seoul, Republic of Korea

**Keywords:** breast cancer, traditional East Asian medicine, survival, recurrence, meta-analysis, systematic review

## Abstract

**Background:** Breast cancer is the most common cancer in women. Patients with cancer increasingly incorporate complementary and alternative medicines, including traditional East Asian medicine (TEAM), for cancer prevention and treatment. This review aimed to determine the effectiveness and safety of TEAM for survival and recurrence after surgery in patients with breast cancer.

**Methods:** We searched nine electronic databases up to 25 August 2022, for randomized controlled trials (RCTs) of TEAM to prevent the recurrence of breast cancer in female patients after mastectomy or breast-conserving surgery. The primary outcome was 5-year disease-free survival (DFS), and secondary outcomes were 5-year overall survival, locoregional and distant recurrence rates, and toxicity. This study adhered to the guidelines of the Preferred Reporting Items for Systematic Reviews and Meta-Analyses (PRISMA). The Grading of Recommendations Assessment, Development, and Evaluation (GRADE) was used to evaluate the quality of evidence.

**Results:** From 368 citations, data from nine studies reporting on a total of 1240 patients were included in the systematic review, and eight studies were deemed suitable for the meta-analysis. TEAM combined with adjuvant chemotherapy showed a significant improvement in DFS (odds ratio [OR] 0.42%, 95% confidence interval [CI] 0.28 to 0.61, *p* < 0.00001) and overall survival (OR 0.44%, 95% CI 0.27 to 0.73, *p* = 0.001) compared to adjuvant chemotherapy alone. The reduction in the rate of total recurrence was favorable for TEAM combined with adjuvant chemotherapy compared to adjuvant chemotherapy alone (Risk ratio 0.49%, 95% CI 0.35 to 0.70; *p* < 0.0001). TEAM after adjuvant chemotherapy showed a significant advantage in DFS compared to no TEAM (OR 0.61%, 95% CI 0.41 to 0.92, *p* = 0.02). No severe adverse events related to TEAM were reported. The overall certainty of the evidence for DFS, overall survival, and the total recurrence rate were moderate when postoperative breast cancer patients used TEAM combined with adjuvant chemotherapy.

**Conclusion:** Moderate-quality evidence suggests TEAM as an add-on therapy to adjuvant chemotherapy. TEAM may have the potential to improve long-term survival and prevent postoperative recurrence in patients with breast cancer. In future, more rigorous RCTs should be conducted to confirm these findings.

## 1 Introduction

Breast cancer is the most commonly diagnosed cancer in women, accounting for 11.7% of the global cancer incidence, and the fifth leading cause of cancer-related mortality worldwide. Over 2.3 million new cases and 685,000 deaths from breast cancer occurred in 2020 (Sung et al., 2021). If the current trends continue, the burden of breast cancer is predicted to increase to over 3 million new cases and 1 million annual deaths by 2040. Global efforts and public health measures are needed to reduce breast cancer mortality by ensuring access to prompt and comprehensive cancer management (Arnold et al., 2022).

Various treatment options are available for patients with breast cancer, including surgery, chemotherapy, radiotherapy, and endocrine therapy (Harbeck and Gnant, 2017). While these conventional therapies can improve survival significantly, a large proportion continues to suffer recurrence, and the quality of patients’ lives is often compromised (Xiang et al., 2019; Berretta et al., 2022). Complementary and alternative medicine is common among patients with cancer; up to 87% have used at least one approach (Judson et al., 2017).

Traditional East Asian medicine (TEAM), as an important component of complementary and alternative medicine, is widely used to enhance the efficacy and manage the side effects of standard cancer therapy and to improve cancer-related symptoms (Wang et al., 2018). It possesses advantages in inhibiting cancer development and recurrence by regulating oncogenes and tumor suppressor genes, remodeling epigenetics, modulating the tumor microenvironment, and eliminating cancer stem cells (Xiang et al., 2019). However, the clinical efficacy and toxicity of TEAM remain a concern to many physicians owing to inconsistent data or a lack of clinical studies.

Some studies reported enhanced tumor response, improved quality of life, and reduced risk of adverse events in patients with breast cancer, on evaluating the effects of TEAM combined with chemotherapy compared with chemotherapy alone. However, long-term survival data were unavailable (Zhu et al., 2016; Lai et al., 2022). Moreover, most reviews did not separately quantify the clinical benefits of TEAM according to the stage or subtype of breast cancer (Wang et al., 2015; Zhu et al., 2016; Ho et al., 2021; Lai et al., 2022). Therefore, this systematic review and meta-analysis aimed to synthesize disease-free and overall survival, local and distant control, and toxicity data to analyze the effectiveness and safety of TEAM, as an add-on therapy to standard adjuvant therapy, on survival and recurrence after surgery in patients with stage I–III breast cancer.

## 2 Methods

The study protocol was prospectively registered on PROSPERO (registration number: CRD42022358887). A systematic literature search was conducted according to the recommendations of the Cochrane Collaboration (Liberati et al., 2009). This systematic review and meta-analysis followed the Preferred Reporting Items for Systematic Reviews and Meta-Analyses (PRISMA) guidelines (Higgins et al., 2022).

### 2.1 Search strategy and study selection

An electronic search was conducted from August 9 to 25 August 2022 using PubMed, Cochrane Library, EMBASE, China National Knowledge Infrastructure (CNKI), Korean databases (KMBASE, KISS, KCI, and OASIS), and a Japanese database (CiNii). There were no restrictions on language and publication date for any of the searches. The medical subject heading (MeSH) terms and text word searching were conducted for each of the following search segments: breast cancer, recurrence, metastasis, post-operation, and traditional East Asian medicine. Details of the search strategies are presented in Supplementary Material S1. All studies were independently screened by two authors according to their titles and abstracts. The full texts of studies considered relevant for the review were obtained to assess eligibility based on the inclusion and exclusion criteria. Any disagreement was resolved *via* discussion, and if necessary, arbitrated by a third researcher.

### 2.2 Inclusion criteria

#### 2.2.1 Types of studies

Randomized controlled trials (RCTs) using parallel group or crossover designs that examined the effect of TEAM in the prevention of postoperative breast cancer recurrence were considered.

#### 2.2.2 Types of participants

Adult female patients (aged ≥18 years) who were diagnosed with stage I–III breast cancer by pathological examination and had no recurrence or metastasis after mastectomy or breast-conserving surgery were included. Studies that did not report stages of cancer were excluded.

#### 2.2.3 Types of interventions

Oral administration of TEAM combined with standard adjuvant therapy (chemotherapy, radiation therapy, and endocrine therapy) for patients with breast cancer or TEAM alone was included as an experimental intervention. Studies were excluded if the composition or dose of herbs could not be found. Control interventions included adjuvant therapy, placebo, no treatment, and usual care. However, studies comparing standard adjuvant therapy with TEAM head-to-head were excluded.

#### 2.2.4 Types of outcome measures

The primary outcome was 5-year disease-free survival (DFS), which is the current gold standard for assessing therapeutic efficacy in patients with breast cancer (Proskurina et al., 2016). The secondary outcomes were 5-year overall survival, locoregional and distant recurrence rates, and toxicity. Studies were excluded if DFS was not reported and the odds ratios (ORs) could not be extrapolated from the available data.

### 2.3 Data extraction

Data were extracted independently by two authors based on a prespecified protocol. The following variables were obtained from each study: study details (first author, publication year, journal, design, and country), study population characteristics (sample size, cancer stage, subtype, and age), intervention and comparison (composition, dose, schedule, duration, and follow-up time), outcome measures, and adverse events. When published data were insufficient, the authors contacted the authors of relevant studies seeking permission to access data to extrapolate ORs.

### 2.4 Risk of bias assessment in included studies

The methodological quality of the included trials was assessed by two authors using Cochrane Collaboration’s risk of bias tool. Bias was categorized into seven domains: random sequence generation, allocation concealment, blinding of participants and personnel, blinding of outcome assessment, incomplete outcome data, selective reporting, and other bias. Each trial was rated as low, high, or unclear for each domain (Higgins et al., 2011). Any disagreement was resolved by discussion between the two authors, and if necessary, a third researcher was consulted.

### 2.5 Data synthesis and statistical analysis

Statistical analysis was performed using Review Manager version 5.4.1 (The Cochrane Collaboration, London, United Kingdom). The OR of 5-year overall survival and DFS were calculated from percentages or the number of events or using digital analysis of the Kaplan-Meier curve. Peto’s method was applied to combine ORs in a meta-analysis of time-to-event outcomes (disease-free and overall survival) (Higgins et al., 2022). The observed minus expected number of events (O – E) and the variance (V) for individual studies were combined across all trials with the fixed-effects model to give a pooled OR (Yusuf et al., 1985). Risk ratios (RRs) were estimated for dichotomous data such as the rate of recurrence and toxicity. The RRs for individual trials were combined across all trials using the Mantel-Haenszel method. The fixed-effects model was used if the number of studies included in the meta-analysis was less than five or if there was no significant heterogeneity. In other cases, a random effects model was used (Tufanaru et al., 2015). The *I* ^*2*^ test was used to assess the study heterogeneity. *I* ^*2*^ values of 25%, 50%, and 75% corresponded to low, intermediate, and high levels of heterogeneity, respectively (Higgins et al., 2003). *p*-value <0.05 was considered statistically significant. If possible, subgroup analysis was performed according to the subtype of breast cancer which is an important factor associated with the survival of breast cancer patients, types of TEAM, or types of chemotherapy. Potential publication bias was assessed by visual inspection of funnel plots when more than 10 studies were included in the meta-analysis.

### 2.6 Quality of evidence

The quality of evidence was assessed according to the Grading of Recommendations Assessment, Development, and Evaluation (GRADE) for each outcome. The grade was classified as high, moderate, low, and very low after considering each of the four key elements: study design, study quality, consistency, and directness (Atkins et al., 2004). A summary table was constructed using GRADEpro (http://gradepro.org/).

## 3 Results

### 3.1 Study selection

**FIGURE 1:**
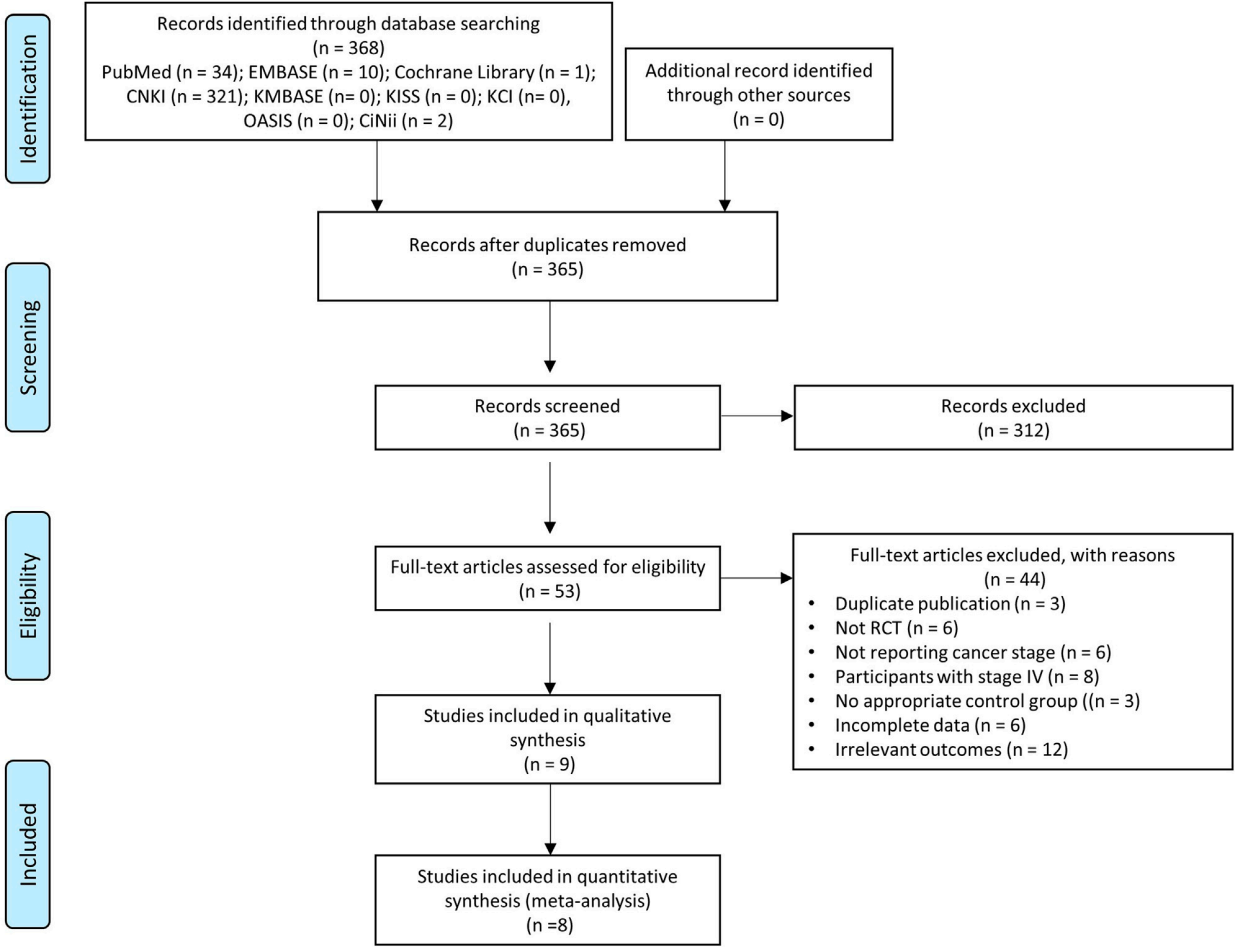
PRISMA flow diagram of study selection.

The search yielded a total of 368 publications. After removing three duplicates from the different databases, irrelevant references (n = 312) were excluded by reviewing the titles and abstracts. A total of 53 studies were identified as possibly relevant and reviewed based on the full text. Of these, 44 studies were excluded: three studies with duplicated data, six studies not RCTs, 14 studies with inappropriate participants, three studies with no appropriate control group, six studies with incomplete data, and 12 studies with irrelevant outcomes. Finally, nine studies were included in the systematic review (Wang et al., 2010; Wei et al., 2012; Yuan et al., 2012; Zhang et al., 2013; Li et al., 2016; Liu et al., 2016; Song and Luo., 2016; Wang et al., 2018; Wu et al., 2022), and eight studies were suitable for the meta-analysis (Wang et al., 2010; Wei et al., 2012; Yuan et al., 2012; Zhang et al., 2013; Li et al., 2016; Liu et al., 2016; Song and Luo., 2016; Wang et al., 2018). The study selection process is summarized in the PRISMA flow diagram (Figure 1).

### 3.2 Study characteristics

All included studies were published in China between 2010 and 2022 (Wang et al., 2010; Wei et al., 2012; Yuan et al., 2012; Zhang et al., 2013; Li et al., 2016; Liu et al., 2016; Song and Luo., 2016; Wang et al., 2018; Wu et al., 2022). A total of 1240 patients were included in the analysis. The mean age of the participants ranged from 39.5 to 52.0 years. Triple-negative breast cancer (TNBC) was the subtype studied by six studies (Wei et al., 2012; Yuan et al., 2012; Zhang et al., 2013; Song and Luo., 2016; Wang et al., 2018; Wu et al., 2022). Among the remaining three studies, one studied hormone receptor (HR)-negative breast cancers (Li et al., 2016), another did not comment on HR and human epidermal growth factor receptor 2 (HER2) status (Liu et al., 2016), and the other focused on estrogen receptor-positive breast cancers (Wang et al., 2010). These studies differed in their interventions. In evaluating the effect of TEAM as an add-on therapy, five studies compared TEAM combined with adjuvant chemotherapy to adjuvant chemotherapy alone (Wang et al., 2010; Yuan et al., 2012; Liu et al., 2016; Song and Luo., 2016; Wang et al., 2018), one study compared TEAM combined with adjuvant chemotherapy to placebo combined with adjuvant chemotherapy (Wu et al., 2022), and three studies compared TEAM to no TEAM after adjuvant chemotherapy (Wei et al., 2012; Zhang et al., 2013; Li et al., 2016). Adjuvant chemotherapy was followed by radiation therapy, endocrine therapy, or no treatment according to the cancer stage and subtype. The duration of TEAM treatment varied between studies. Three studies treated patients for 1 year or less (Li et al., 2016; Liu et al., 2016; Wang et al., 2018), and the other six studies treated patients for 2 years or more (Wang et al., 2010; Wei et al., 2012; Yuan et al., 2012; Zhang et al., 2013; Song and Luo., 2016; Wu et al., 2022), ranging from 6 weeks to 5 years. DFS was reported in all studies (Wang et al., 2010; Wei et al., 2012; Yuan et al., 2012; Zhang et al., 2013; Li et al., 2016; Liu et al., 2016; Song and Luo., 2016; Wang et al., 2018; Wu et al., 2022); overall survival was assessed in five studies (Li et al., 2016; Liu et al., 2016; Song and Luo., 2016; Wang et al., 2018; Wu et al., 2022); and the recurrence rate was used as an outcome measure in six studies (Wang et al., 2010; Yuan et al., 2012; Li et al., 2016; Liu et al., 2016; Wang et al., 2018; Wu et al., 2022). The study characteristics are presented in Table 1.

**TABLE 1:** Characteristics of the included studies.

Study	Sample size	Mean age (E/C)	ER/PR/HER2 subtypes	Cancer stage	Experimental intervention (CTx regimen, if reported)	Control intervention	Duration of TEAM	Outcome
Liu et al. (2016)	100	51.84/51.14	NR	Ⅰ–Ⅲ	Ginsenoside capsule, berberine tablet + CTx (FEC)	CTx	6 weeks	1. RFS
								2. KPS
								3. Levels of tumor markers
								4. LRR
								5. DRR
								6. OS
								7. Median survival time
Song and Luo. (2016)	100	45.0/42.0	TNBC	Ⅰ–Ⅱ	Chaihushugan decoction	CTx	≥3.5 years	1. DFS
					+ CTx (AC-T)			2. OS
								3. Death rate
Wang et al. (2018a)	168	48.5/48.7	TNBC	Ⅰ–Ⅲ	Shenghe powder + CTx (NR)	CTx	1 year	1. DFS
								2. OS
								3. LRR
								4. DRR
								5. Levels of immune cells
								6. miR-34a, LD
Wang Q et al. (2010)	103	47.0	+/NR/NR	Ⅰ–Ⅲ	Fuzheng Xiaoliu decoction	CTx	≥2.5 years	1. DFS
					+ CTx (NR)			2. TRR
								3. Levels of CBC
								4. FACT-B
Yuan et al. (2012)	69	47.0	TNBC	Ⅱ–Ⅲ	Rufufang + CTx (GP)	CTx	5 years	1. DFS
								2. TRR
Wu et al. (2022)	252	52.0/51.0	TNBC	Ⅰ–Ⅲ	Sanyin formula + CTx (various^*^)	Placebo + CTx	≥2 years	1. DFS
								2. OS
								3. LRR
								4. DRR
Li et al. (2016)	358	47.2/47.5	−/−/+	Ⅰ–Ⅲ	Huaier granule after CTx	No TEAM after CTx	3–6 months	1.DFS
								2. TRR
								3. OS
								4. BCFI
								5. QoL
Wei et al. (2012)	48	39.5/40.3	TNBC	Ⅱ–Ⅲ	Compound Ban-mao capsule after CTx	No TEAM after CTx	25 months	1. DFS
								2. Total effective rate
Zhang et al. (2013)	42	48.0	TNBC	Ⅱ–Ⅲ	Modified Compound Ban-mao capsule and Hong-dou-shan formula after CTx	No TEAM after CTx	30 months	1. DFS
								2. Total effective rate

Abbreviations: A, doxorubicin; BCFI, breast cancer-free interval; C, cyclophosphamide; CTx, adjuvant chemotherapy; DFS, disease-free survival; DRR, distant recurrence rate; E, epirubicin; E/C, Experimental/Control; ER, estrogen receptor; F, fluorouracil; FACT-B, Functional Assessment of Cancer Therapy-Breast Cancer; G, gemcitabine; GI, gastrointestinal; HER2, human epidermal growth factor receptor 2; IHC, immunohistochemistry; KPS, karnofsky performance scale; LD, L-lactate dehydrogenase; LRR, locoregional recurrence rate; miR-34a, microRNA, 34a; NR, not reported; P, cisplatin; PFS, progression-free survival; PR, progesterone receptor; QoL, quality of life; RFS, recurrence-free survival; T, taxane (docetaxel or paclitaxel); TEAM, traditional East Asian medicine; TNBC, triple-negative breast cancer; TRR, total recurrence rate (LRR, plus DRR).

*EC-T, EC, CEF-T, AC-T, or other chemotherapy regimens were used in the study.

The TEAM in the included studies is shown in Supplementary Material S2. Various forms of herbs were administered to patients as follows: single herbs, herbal formulas (a combination of several herbs), and herb-derived phytochemicals. Among the herbal components with the highest composition ratio in each study, primarily associated with the traditional use of the herbal formula, *Scutellaria barbata* D. Don (*S. barbata*) was the most common and was used in four studies (Wei et al., 2012; Yuan et al., 2012; Zhang et al., 2013; Wu et al., 2022). *Hedyotis diffusa* Willd. (*H. diffusa*) was the next most frequently used herb, which was used in two studies (Yuan et al., 2012; Wang et al., 2018).

### 3.3 Risk of bias assessment of the included studies

**FIGURE 2:**
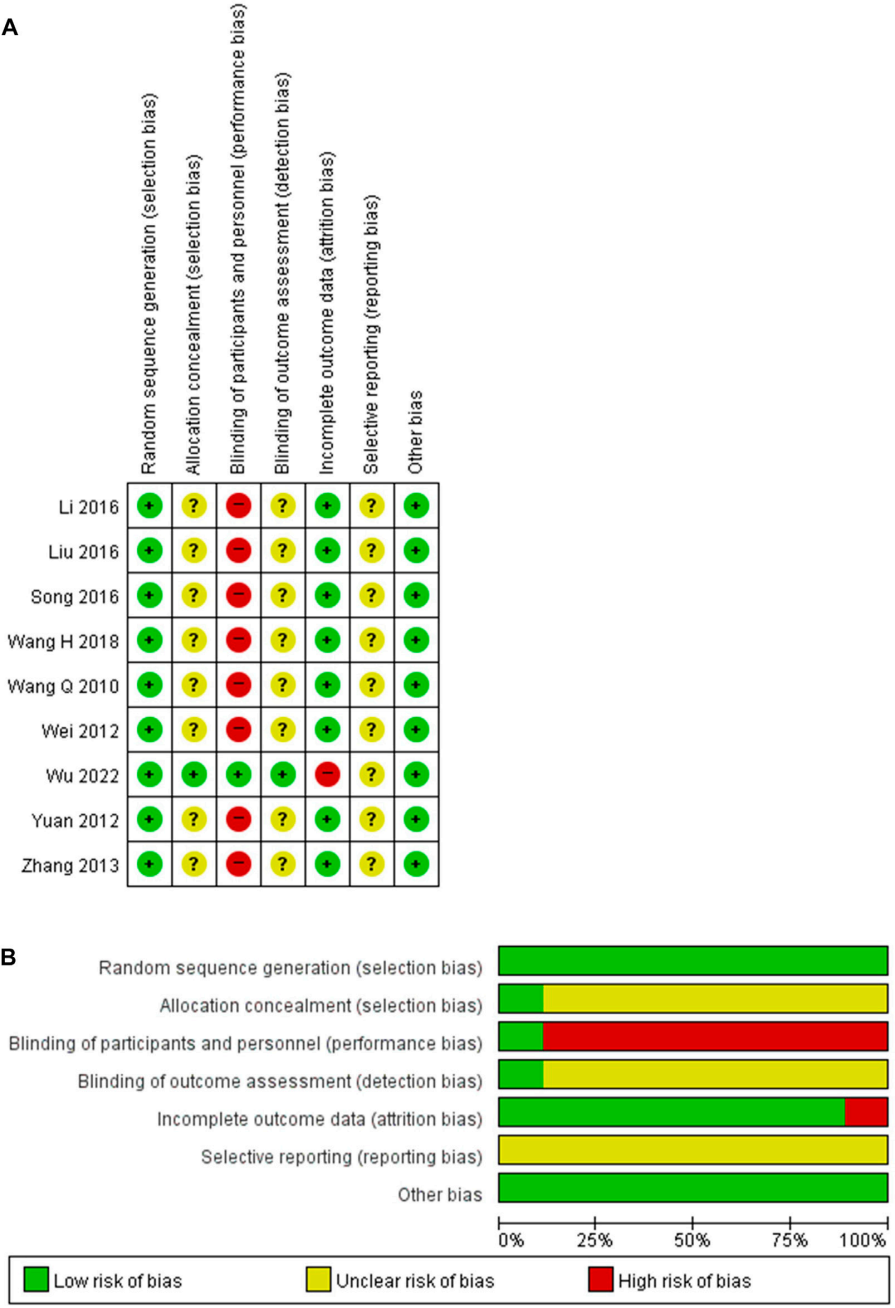
Risk of bias for the included studies. **(A)** Risk of bias summary. **(B)** Risk of bias graph.+, low risk of bias; ?, unclear of bias; −, high risk of bias.

Random sequence generation was judged as adequate in all studies, and the risk of bias was assessed as low. While the adequacy of allocation concealment could not be evaluated for the last studies (Wang et al., 2010; Wei et al., 2012; Yuan et al., 2012; Zhang et al., 2013; Li et al., 2016; Liu et al., 2016; Song and Luo., 2016; Wang et al., 2018), only one study that implemented a central randomization system was considered to have a low risk of bias (Wu et al., 2022). The blinding of participants and personnel was adequately described in one study, and the risk of bias was assessed to be low (Wu et al., 2022). However, the remaining studies without a placebo were determined to have a high risk of bias (Wang et al., 2010; Wei et al., 2012; Yuan et al., 2012; Zhang et al., 2013; Li et al., 2016; Liu et al., 2016; Song and Luo., 2016; Wang et al., 2018). One study blinded the assessor to the exposure status and was evaluated as having a low risk of bias (Wu et al., 2022). The others provided insufficient data and could not be assessed in the blinding of outcome assessment (Wang et al., 2010; Wei et al., 2012; Yuan et al., 2012; Zhang et al., 2013; Li et al., 2016; Liu et al., 2016; Song and Luo., 2016; Wang et al., 2018). Even though the results were analyzed in intention-to-treat, one study reporting a dropout rate of 23% was judged to have a high risk of bias due to incomplete data (Wu et al., 2022). Another study with a dropout rate of less than 10% revealed a low risk of bias (Yuan et al., 2012). The others without dropouts were also determined to have a low risk of bias (Wang et al., 2010; Wei et al., 2012; Zhang et al., 2013; Li et al., 2016; Liu et al., 2016; Song and Luo., 2016; Wang et al., 2018). All studies were evaluated as having an uncertain risk of bias in selective reporting and a low risk of bias in other bias. The risk of bias in the included studies is shown in Figure 2.

### 3.4 Effectiveness of TEAM as an add-on therapy

#### 3.4.1 Disease-free survival

**FIGURE 3:**
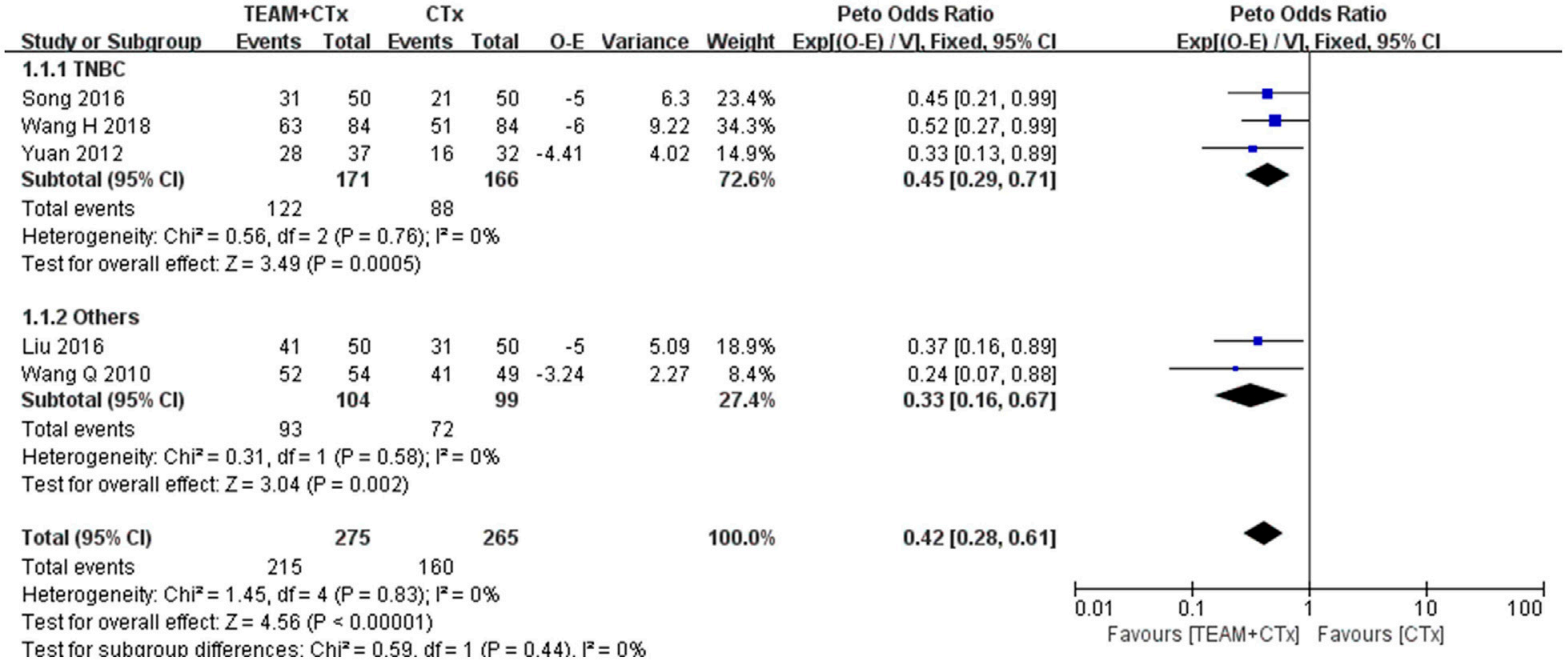
Odds ratio of disease-free survival in breast cancer patients (TEAM plus adjuvant chemotherapy *versus* adjuvant chemotherapy alone). O – E, observed minus expected number of events; CTx, adjuvant chemotherapy; TEAM, traditional East Asian medicine; TNBC, triple-negative breast cancer; V, variance.

To evaluate the effectiveness of TEAM combined with adjuvant chemotherapy, five studies reporting sufficient data to estimate the OR for DFS were included in the meta-analysis (Wang et al., 2010; Yuan et al., 2012; Liu et al., 2016; Song and Luo., 2016; Wang et al., 2018). There was a statistically significant benefit for DFS with TEAM combined with adjuvant chemotherapy compared with adjuvant chemotherapy alone (OR 0.42%, 95% confidence interval [CI] 0.28 to 0.61, *p* < 0.00001) without any evidence of statistical heterogeneity (*I* ^*2*^ = 0%). A subgroup analysis was performed on TNBC patients, and three studies were eligible for inclusion (Yuan et al., 2012; Song and Luo., 2016; Wang et al., 2018). The odds of DFS for the TNBC studies favored TEAM plus adjuvant chemotherapy over adjuvant chemotherapy alone (OR 0.45%, 95% CI 0.29 to 0.71; *p* = 0.0005). No evidence of heterogeneity was found (*I* ^*2*^ = 0%) (Figure 3).

**FIGURE 4:**
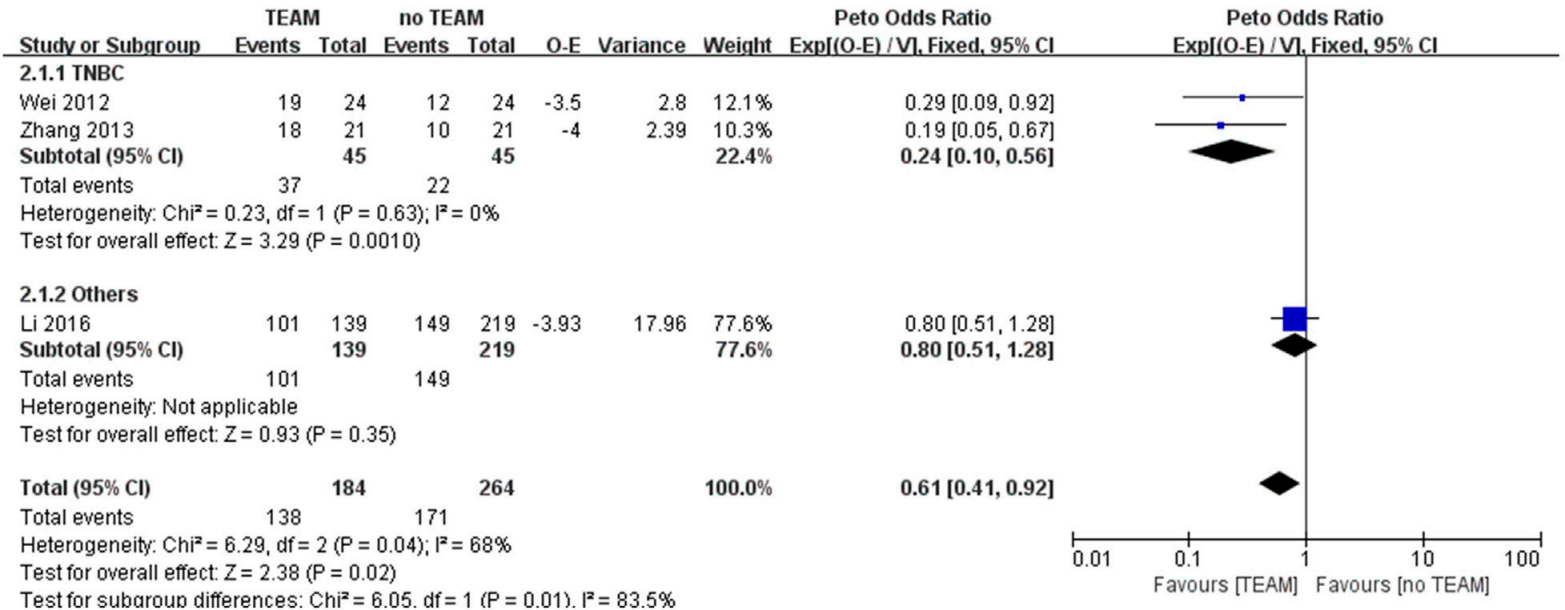
Odds ratio of disease-free survival in breast cancer patients with TEAM *versus* no TEAM after adjuvant chemotherapy. O – E, observed minus expected number of events; TEAM, traditional East Asian medicine; TNBC, triple-negative breast cancer; V, variance.

To evaluate the effectiveness of TEAM after adjuvant chemotherapy, three studies were included in the meta-analysis (Wei et al., 2012; Zhang et al., 2013; Li et al., 2016). There was a significant advantage in DFS with TEAM compared to no TEAM after adjuvant chemotherapy (OR 0.61%, 95% CI 0.41 to 0.92; *p* = 0.02) with considerable heterogeneity (*I* ^*2*^ = 68%). The subgroup analysis of TNBC patients strongly favored TEAM over no TEAM after adjuvant chemotherapy (OR 0.24%, 95% CI 0.10 to 0.56; *p* = 0.001) with no heterogeneity (Figure 4).

#### 3.4.2 Overall survival

**FIGURE 5:**
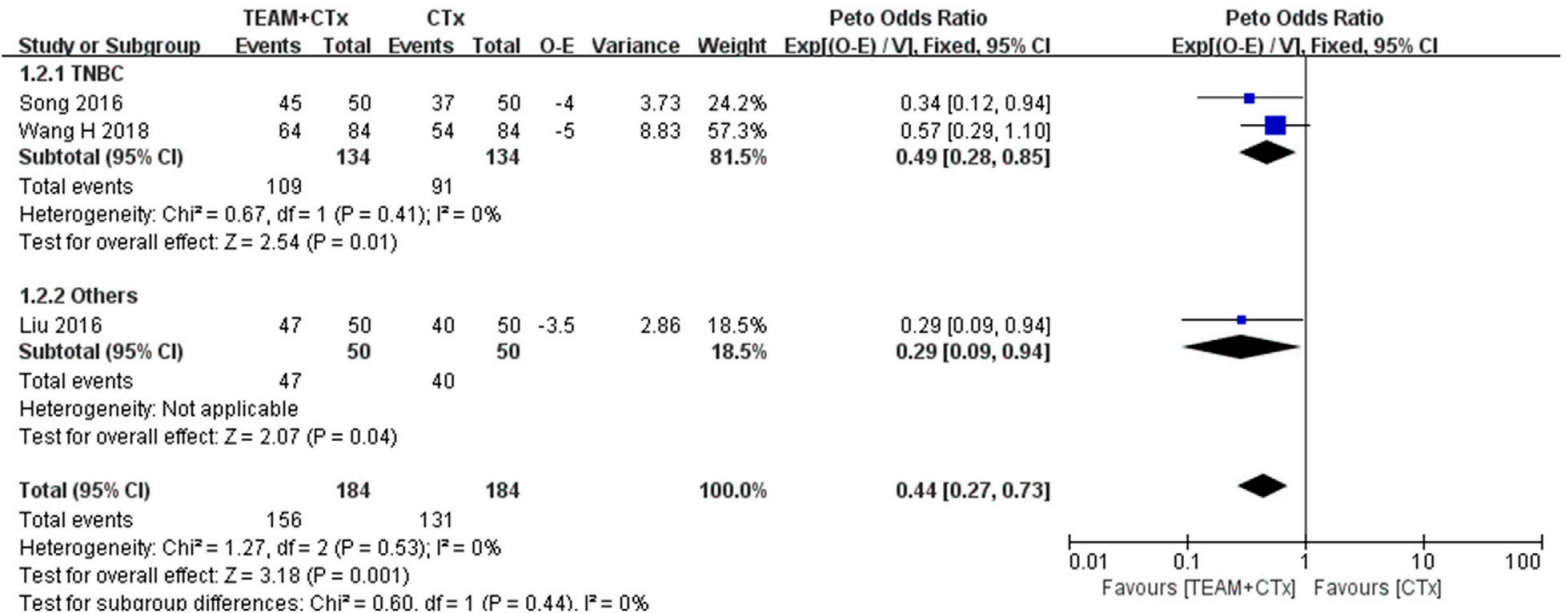
Odds ratio of overall survival in breast cancer patients (TEAM plus adjuvant chemotherapy *versus* adjuvant chemotherapy alone). O – E, observed minus expected number of events; CTx, adjuvant chemotherapy; TEAM, traditional East Asian medicine; TNBC, triple-negative breast cancer; V, variance.

Three studies reporting data available to calculate OR for overall survival were eligible for inclusion in the meta-analysis (Liu et al., 2016; Song and Luo., 2016; Wang et al., 2018). TEAM combined with adjuvant chemotherapy was significantly associated with an improvement in overall survival (OR 0.44%, 95% CI 0.27 to 0.73; *p* = 0.001) with no heterogeneity. The pooled OR estimate for the TNBC studies showed a significant difference favoring TEAM plus adjuvant chemotherapy over adjuvant chemotherapy alone (OR 0.49%, 95% CI 0.28 to 0.85, *p* = 0.01) (Song and Luo., 2016; Wang et al., 2018). No evidence of heterogeneity was found between studies (*I* ^*2*^ = 0%) (Figure 5).

#### 3.4.3 Locoregional and distant recurrence rate

**FIGURE 6:**
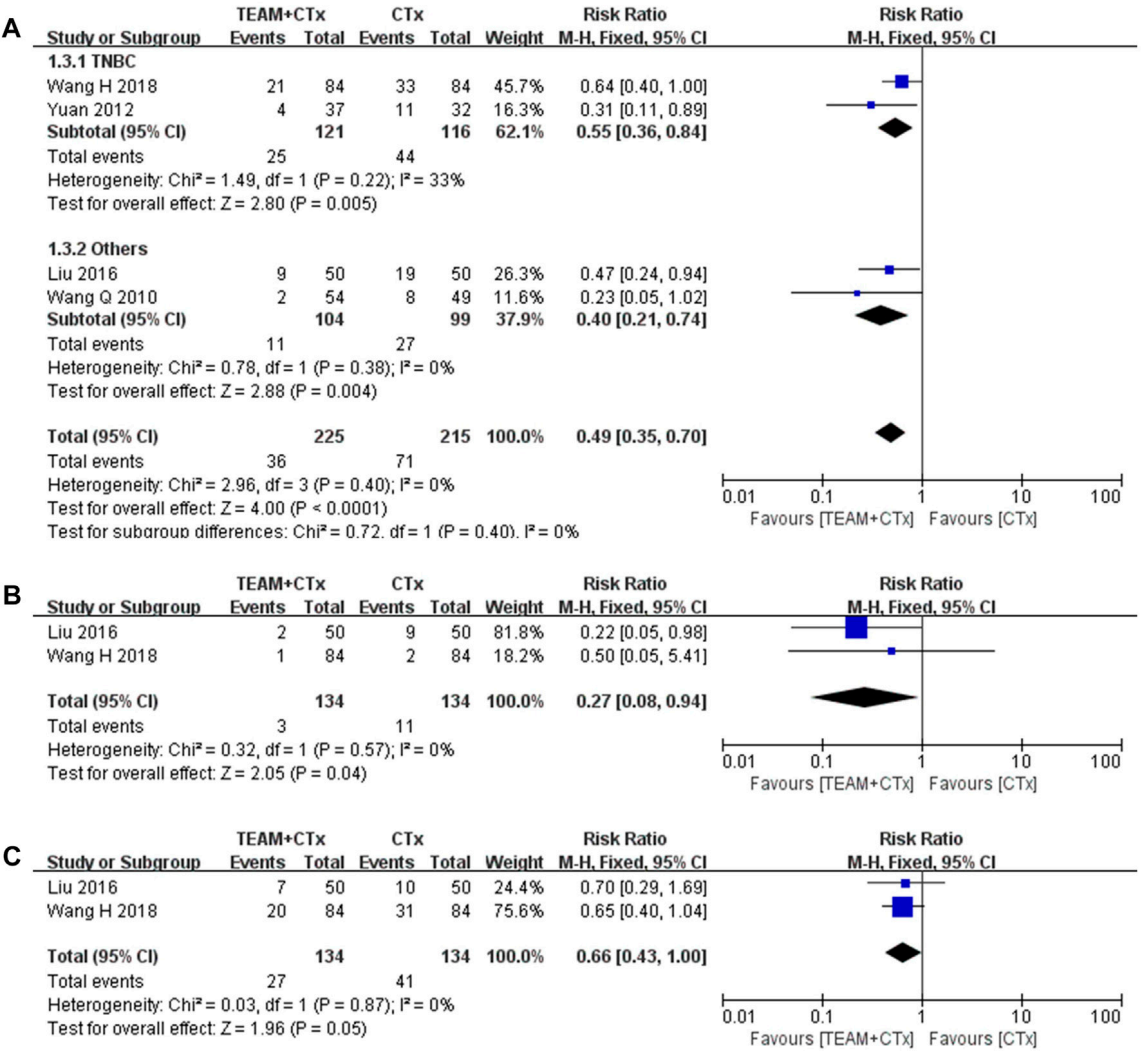
Risk ratios of rates of locoregional and distant recurrence in breast cancer patients (TEAM plus adjuvant chemotherapy *versus* adjuvant chemotherapy alone). **(A)** Total recurrence rate. **(B)** Locoregional recurrence rate. **(C)** Distant recurrence rate.CTx, adjuvant chemotherapy; TEAM, traditional East Asian medicine; TNBC, triple-negative breast cancer.

Four studies reporting the rate of total recurrence were eligible for the meta-analysis of RR (Wang et al., 2010; Yuan et al., 2012; Liu et al., 2016; Wang et al., 2018). These data showed a significant reduction in the occurrence of total recurrence with TEAM plus adjuvant chemotherapy, overall (RR 0.49%, 95% CI 0.35 to 0.70; *p* < 0.0001) with no heterogeneity and in TNBC studies (RR 0.55, 95% CI 0.36 to 0.84, *p* = 0.005) with intermediate heterogeneity (*I* ^*2*^ = 33%) (Figure 6A). In addition, two out of four studies reporting locoregional and distant recurrence rates separately were meta-analyses (Liu et al., 2016; Wang et al., 2018). The rate of locoregional recurrence was significantly reduced by TEAM plus adjuvant chemotherapy compared with adjuvant chemotherapy alone (RR 0.27%, 95% CI 0.08 to 0.94; *p* = 0.04) with no heterogeneity (Figure 6B). However, the rate of distant recurrence did not differ significantly (RR 0.66%, 95% CI 0.43 to 1.00; *p* = 0.05), with no heterogeneity (Figure 6C).

### 3.5 Adverse events

Two of nine studies reported adverse events (Liu et al., 2016; Wu et al., 2022). One study comparing TEAM combined with adjuvant chemotherapy *versus* adjuvant chemotherapy alone reported no significant difference in hematological toxicity (white cell count, 17.0% vs. 17.5%) and gastrointestinal toxicity (nausea and vomiting, 10.6% vs. 15%; diarrhea, 6.4% vs. 7.4; anorexia, 14.9% vs. 15%) (Liu et al., 2016). In another study, upper limb edema, pain, and diarrhea were observed in the TEAM combined with adjuvant chemotherapy group, whereas abnormalities in liver function, weakness, pain, and diarrhea were reported in placebo combined with adjuvant therapy, with no significant difference in the rate of adverse events between the groups (2.4% vs. 3.2%) (Wu et al., 2022). No treatment-related deaths or life-threatening events occurred in either study.

### 3.6 Quality of evidence

In the comparison of TEAM plus adjuvant chemotherapy with adjuvant chemotherapy alone, the certainty of the evidence was moderate for disease-free and overall survival, and total recurrence rates. However, the certainty of the evidence was low for locoregional and distant recurrence rates. In the comparison of TEAM with no TEAM after adjuvant therapy, the certainty of the evidence for DFS was low. The certainty of the evidence for all outcomes was downgraded mainly due to serious study limitations and the small sample size (Table 2).

**TABLE 2:** Summary of findings.

TEAM as an add-on therapy on survival and recurrence after surgery for breast cancer					
**Patient or population:** Postoperative breast cancer patients					
**Intervention:** TEAM combined with adjuvant chemotherapy					
**Comparison:** Adjuvant chemotherapy					
**Outcomes**	Anticipated absolute effects* (95% CI)		**Relative effect (95% CI)**	**No. Of participants (studies)**	**Certainty of the evidence (GRADE)**
	**Risk with control**	**Risk with intervention**			
Disease-free survival	604 per 1,000	**254 per 1,000** (169–368)	**Peto OR 0.42** (0.28–0.61)	540 (5 RCTs)	⊕⊕⊕○
					MODERATE
Overall survival	712 per 1,000	**313 per 1,000** (192–520)	**Peto OR 0.44** (0.27–0.73)	368 (3 RCTs)	⊕⊕⊕○
					MODERATE
Total recurrence rate	330 per 1,000	**162 per 1,000** (116–231)	**RR 0.49** (0.35–0.70)	440 (4 RCTs)	⊕⊕⊕○
					MODERATE
Locoregional recurrence rate	82 per 1,000	**22 per 1,000** (7–77)	**RR 0.27** (0.08–0.94)	268 (2 RCTs)	⊕⊕○○
					LOW
Distant recurrence rate	306 per 1,000	**202 per 1,000** (132–306)	**RR 0.66** (0.43–1.00)	268 (2 RCTs)	⊕⊕○○
					LOW
**Patient or population:** Postoperative breast cancer patients					
**Intervention:** TEAM after adjuvant chemotherapy					
**Comparison:** No TEAM after adjuvant chemotherapy					
**Outcomes**	Anticipated absolute effects* (95% CI)		**Relative effect (95% CI)**	**No. Of participants (studies)**	**Certainty of the evidence (GRADE)**
	**Risk with control**	**Risk with intervention**			
Disease-free survival	648 per 1,000	**395 per 1,000** (266–596)	**Peto OR 0.61** (0.41–0.92)	448 (3 RCTs)	⊕⊕○○
					LOW

*The risk in the intervention group (and its 95% confidence interval) is based on the assumed risk in the comparison group and the relative effect of the intervention (and its 95% CI). CI, confidence interval; No, number; OR, odds ratio; RCT, randomized controlled study; RR, risk ratio; TEAM, traditional East Asian medicine. GRADE Working Group grades of evidence. High certainty: We are very confident that the true effect lies close to that of the estimate of the effect. Moderate certainty: We are moderately confident in the effect estimate: The true effect is likely to be close to the estimate of the effect, but there is a possibility that it is substantially different. Low certainty: Our confidence in the effect estimate is limited: The true effect may be substantially different from the estimate of the effect. Very low certainty: We have very little confidence in the effect estimate: The true effect is likely to be substantially different from the estimate of effect.

## 4 Discussion

In this systematic review and meta-analysis, TEAM as an add-on therapy to standard adjuvant therapy was identified as potentially exhibiting a positive impact on improving long-term survival and preventing postoperative recurrence in patients with breast cancer. In addition, patients with triple-negative tumors with a poorer prognosis than those of other subtypes could benefit from the use of TEAM. TEAM combined with adjuvant chemotherapy was associated with a better DFS than adjuvant chemotherapy alone. Subgroup analysis of TNBC studies found that TEAM combined with adjuvant chemotherapy was associated with favorable outcomes in DFS compared to adjuvant therapy alone. Moreover, there was a significant improvement in DFS with TEAM compared with no TEAM after adjuvant chemotherapy in HR-negative and TNBC breast cancers who do not benefit from endocrine therapy. In secondary outcomes, overall survival was longer in the TEAM combined with adjuvant chemotherapy group than in the adjuvant chemotherapy group, in accordance with the findings of the subgroup analysis of TNBC studies. A significant reduction in the total recurrence rate was associated with TEAM combined with adjuvant chemotherapy compared with that associated with adjuvant therapy alone. The overall quality of evidence for DFS, overall survival, and the total recurrence rate was moderate when postoperative breast cancer patients used TEAM combined with adjuvant chemotherapy.

According to the traditional view, it is believed that TEAM posseses advantages in symptom management at the terminal stage when conventional medicine cannot offer any other treatment options. However, recent evidence proposes that TEAM used in different stages of cancer lesions might be beneficial to the entire course of cancer prevention and treatment, including recovery from post-operation, and when undergoing radiotherapy or chemotherapy (Qi et al., 2015). Previous studies on the effect of TEAM combined with conventional cancer treatment on postoperative recurrence and survival have been reported in non-small cell lung cancer (Kawai and Saito, 2020), hepatocellular carcinoma (Zhao et al., 2020), bladder cancer (Chen et al., 2014), and breast cancer (Wang et al., 2015; Zhu et al., 2016; Ho et al., 2021; Lai et al., 2022). A phase IB trial conducted in the United States reported that an aqueous extract of *S. barbata* was well tolerated and showed promising clinical evidence of anticancer activity in patients with metastatic breast cancer (Perez et al., 2010). In addition, a phase IIA trial conducted in Taiwan (Kuo et al., 2012) suggested that the use of the herbal formula mainly composed of *H. diffusa* appears to be a safe alternative adjuvant treatment for patients with refractory metastatic breast cancer. Our findings on the effectiveness of TEAM combined with chemotherapy on survival and recurrence in patients with breast cancer were comparable to those of previous studies.

From the perspective of traditional oriental medicine, cancer originates from qi stagnation, which can diminish local blood circulation and create localized hypoxic conditions that may promote inflammation and tumor growth (Yoon et al., 2014). The main use of TEAM in the included studies, used after adjuvant chemotherapy, was clearing heat, detoxifying, and dispersing blood stasis to activate the immune system and maintain stable disease. Patients receiving standard cancer therapy (e.g., surgery, chemotherapy, and radiation therapy) may experience syndromes such as dual deficiency of qi and yin (Wang et al., 2010). The main use of TEAM in the included trials, combined with adjuvant chemotherapy, was tonifying qi and yin-blood to enhance the efficacy and reduce the side effects of the standard cancer therapy. Our findings on the three main traditional uses of TEAM with standard cancer therapy–clearing heat and detoxifying, removing blood stasis, and tonifying qi and yin-blood–supported the claims of the previous review (Wang H. et al., 2018).

*S. barbata* and *H. diffusa* were found to be the first and second most frequently used herbs among the herbs with the highest composition ratio in the included trials. This result is consistent with that of a previous population-based study conducted in Taiwan, which showed *S. barbata* plus *H. diffusa* was the most common herb pair used for the core treatment of breast cancer (Yeh et al., 2014). The *S. barbata* extract is selectively cytotoxic to breast cancer cells while sparing normal cells by inducing high levels of reactive oxygen species and severe DNA damage (Fong et al., 2008). Neo-clerodane diterpenoids extracted from *S. barbata* demonstrated anticancer activity in multidrug-resistant breast cancer cells *via* inhibition of P-glycoprotein (Xue et al., 2016). Methylanthraquinone from *H. diffusa* induced apoptosis in breast cancer cells *via* an increase in intracellular calcium levels, calpain activation, and caspase-4 cleavage (Liu et al., 2010). Two anthraquinones from *H. diffusa* showed inhibitory activity against Src protein tyrosine kinase, which might account for their potency to induce growth arrest and apoptosis (Shi et al., 2008).

To our knowledge, this is the first systematic review to examine the effectiveness and safety of TEAM for survival and recurrence after surgery in patients with stage I–III breast cancer. The findings are based on a thorough and up-to-date literature search by applying GRADE to judge the quality of evidence. This review had several limitations. All included trials were conducted in China, where favorable cultural factors exist, such as expectations and beliefs regarding TEAM benefits. The effects of TEAM observed in China may differ from those reported in western countries. Another drawback is that inter-study heterogeneity was significant, including differences in the components of TEAM, treatment duration, and chemotherapy regimen. Regarding methodological quality, studies generally included the presence of performance and detection bias, whereas only one study used a placebo. A meta-analysis of the safety of TEAM could not be performed because only two studies reported adverse events. Thus, the findings of this review should be cautiously extrapolated to patients.

## 5 Conclusion

For patients with breast cancer, moderate-quality evidence suggests that TEAM combined with adjuvant chemotherapy is effective in improving long-term survival and reducing postoperative recurrence. The administration of TEAM with adjuvant chemotherapy was not associated with any increased risk of toxicity or severe adverse events. Higher-quality RCTs with larger sample sizes are required to confirm the effectiveness and safety of TEAM, with emphasis on commonly used herbs for patients with breast cancer, such as *S. barbata* and *H. diffusa.*

## Data Availability

The original contributions presented in the study are included in the article/Supplementary Material, further inquiries can be directed to the corresponding author.
