# Supplementary material for: Impact of traditional East Asian medicine as an add-on therapy on survival and recurrence after surgery for breast cancer: A systematic review and meta-analysis
Source: Front Pharmacol. 2023 Apr 4;14:1125373. doi: 10.3389/fphar.2023.1125373 (PMC10110841; doi:10.3389/fphar.2023.1125373)
Supplement: Supplementary file 1 [file DataSheet2.docx]

Supplementary Material

Impact of Traditional East Asian Medicine as an Add-on Therapy on Survival and Recurrence after Surgery for Breast Cancer: A Systematic Review and Meta-Analysis

Jee-hyun Yoon, Eun Hye Kim, Su Bin Park, Hayun Jin, Seong Woo Yoon*

*** Correspondence:** Seong Woo Yoon: stepano212@hanmail.net

# Supplementary material 2. Traditional East Asian medicine in the included studies

| Study | Prescription | Traditional use | Dose | Component |
| --- | --- | --- | --- | --- |
| Li et al. (2016) | Huaier Granule | Heat-clearing and  blood-cooling | Powered herb 20g tid | Fruiting body of *Poria robiniophila* (Murrill) Ginns, 20g |
| Liu et al. (2016) | Ginsenoside Capsule  Berberine Tablet | N/A | 25mg 1capsule qd  0.1g 1tablet qd | Root of *Panax ginseng* C.A.Mey.  Rhizome of *Coptis chinensis* Franch. |
| Song et al. (2016) | Chaihushugan Decoction | Soothing the liver and regulating qi | ½ of extract bid | Rhizome of *Ligusticum chuanxiong* Hort., 20g  Fruiting body of *Coriolus versicolor* (L. ex Fr.) Quel, 20g  Root of *Bupleurum chinense* DC., 15g  Root of *Paeonia lactiflora* Pall., 15g  Unripe fruit of *Citrus aurantium* L., 15g  Peel of ripe fruit of *Citrus reticulata* Blanco, 15g  Herb of *Solanum lyratum* Thunb., 15g  Herb of *Hedyotis diffusa* Willd., 15g  Rhizome of *Cyperus rotundus* L., 10g  Root and rhizome of *Glycyrrhiza uralensis* Fisch., 10g |
| Wang H et al. (2018) | Shenghe Powder | 1. Tonifying qi and replenishing blood  2. Regulating qi and detoxifying  3. Dispelling phlegm and blood stasis | Dry extract qd | Herb of *Hedyotis diffusa* Willd., 20g  Root of *Codonopsis pilosula* (Franch.) Nannf., 15g  Rhizome of *Atractylodes macrocephala* Koidz., 15g  Fruit of *Trichosanthes kirilowii* Maxim., 12g  Stem of *Spatholobus suberectus* Dunn, 12g  Root of *Rehmannia glutinosa* Libosch., 9g  Rhizome of *Curcuma phaeocaulis* Val., 6g  Fruit of *Psoralea corylifolia* L., 6g  Root and rhizome of *Glycyrrhiza uralensis* Fisch., 6g  Fruit of *Amomum villosum* Lour., 3g |
| Wang Q et al. (2010) | Fuzheng Xiaoliu Decoction | 1. Replenishing qi and nourishing yin  2. Clearing heat and detoxifying  3. Promoting blood circulation and removing blood stasis | ½ of extract bid,  or ⅓ of extract tid | Root of *Astragalus mongholicus* Bunge, 30g  Sclerotium of *Polyporus umbellatus* (Pers.) Fires, 30g  Seed of *Coix lacryma-jobi* L. var. *ma-yuen* (Roman.) Stapf, 30g  Bulb of *Lilium lancifolium* Thunb., 30g  Herb of *Hedyotis diffusa* Willd., 15g  Herb of *Scutellaria barbata* D.Don, 15g  Aerial part of *Agrimonia pilosa* Ledeb., 15g  Fruiting body of *Ganoderma lucidum* (Leyss. ex. Fr.) Karst., 12g  Pseudobulb of *Cremastra appendiculata* (D.Don) Makino, 12g  Rhizome of *Sparganium stoloniferum* Buch.-Ham., 10g  Rhizome of *Curcuma phaeocaulis* Val., 10g  Tuber of *Dioscorea bulbifera* L., 10g  Prepared tuber of *Pinellia ternata* (Thunb.) Breit., 10g  Root of *Panax quinquefolium* L., 9g  Peel of ripe fruit of *Citrus reticulata* Blanco, 6g  Root and rhizome of *Glycyrrhiza uralensis* Fisch., 6g |
| Wei et al. (2012) | Compound Banmao Capsule | 1. Clearing heat and detoxifying  2. Dispersing blood stasis  3. Reinforcing the healthy qi | 3 capsules (powdered whole herbs 0.25g) bid | Herb of *Scutellaria barbata* D.Don, 357g  Root of *Astragalus mongholicus* Bunge, 297.5g  Root and rhizome of *Acanthopanax senticosus* (Rupr. et Maxim.) Harms, 297.5g  Pulp of *Cornus officinalis* Sieb. et Zucc., 119g  Fruit of *Ligustrum lucidum* Ait., 119g  Rhizome of *Sparganium stoloniferum* Buch.-Ham., 95g  Rhizome of *Curcuma phaeocaulis* Val., 95g  Root and rhizome of *Panax ginseng* C.A.Mey., 59.5g  Root and rhizome of *Glycyrrhiza uralensis* Fisch., 59.5g  Body of *Mylabris phalerata* Pallas, 23.8g  Bile obtained from *Selenaretos thibetanus* Cuvier, 2.4g |
| Wu et al. (2022) | Sanyin Formula | 1. Tonifying qi and nourishing yin  2. Clearing heat and detoxifying  3. Promoting blood circulation and removing blood stasis | 2 bags (Dry extract 7g) bid | Root of *Codonopsis pilosula* (Franch.) Nannf., 12g  Rhizome of *Atractylodes macrocephala* Koidz, 9g  Sclerotium of *Poria cocos* (Schw.) Wolf, 15g  Herb of *Salvia chinensis* Benth., 30g  Rhizome of *Curcuma phaeocaulis* Val., 30g  Leaf of *Epimedium brevicornu* Maxim., 15g  Aerial part of *Solanum nigrum* L., 30g  Herb of *Scutellaria barbata* D.Don, 30g  Spike of *Prunella vulgaris* L., 9g (total 180g) |
| Yuan et al. (2012) | Rufufang | 1. Tonifying qi and nourishing yin  2. Soothing the liver and detoxifying  3. Promoting blood circulation and removing blood stasis | ½ of extract bid | Herb of *Scutellaria barbata* D.Don, 20g  Herb of *Hedyotis diffusa* Willd., 20g  Seed of *Cuscuta australis* R.Br., 20g  Fruit of *Lycium barbarum* L., 20g  Sclerotium of *Poria cocos* (Schw.) Wolf, 15g  Rhizome of *Atractylodes macrocephala* Koidz., 15g  Rhizome of *Dioscorea opposita* Thunb., 15g  Fruit of *Psoralea corylifolia* L., 15g  Root and rhizome of *Panax ginseng* C.A.Mey., 10g  Fruiting body of *Ganoderma lucidum* (Leyss. ex. Fr.) Karst., 10g  Pseudobulb of *Cremastra appendiculata* (D.Don) Makino, 10g  Rhizome of *Cyperus rotundus* L., 10g  Root of *Angelica sinensis* (Oliv.) Diels, 10g  Peeled root of *Paeonia lactiflora* Pall., 10g  Herb of *Taraxacum mongolicum* Hand.-Mazz., 10g |
| Zhang et al. (2013) | 1) Compound Banmao Capsule  2) Modified Compound Hongdoushan Formula | 1. Clearing heat and detoxifying  2. Dispersing blood stasis  3. Reinforcing the healthy qi | 1) 3 capsules (powdered whole herbs) tid  2) ½ of extract bid | 1) Compound Banmao Capsule  Herb of *Scutellaria barbata* D.Don, 357g  Root of *Astragalus mongholicus* Bunge, 297.5g  Root and rhizome of *Acanthopanax senticosus* (Rupr. et Maxim.) Harms, 297.5g  Pulp of *Cornus officinalis* Sieb. et Zucc., 119g  Fruit of *Ligustrum lucidum* Ait., 119g  Rhizome of *Sparganium stoloniferum* Buch.-Ham., 95g  Rhizome of *Curcuma phaeocaulis* Val., 95g  Root and rhizome of *Panax ginseng* C.A.Mey., 59.5g  Root and rhizome of *Glycyrrhiza uralensis* Fisch., 59.5g  Body of *Mylabris phalerata* Pallas, 23.8g  Bile obtained from *Selenaretos thibetanus* Cuvier, 2.4g  2) Modified Compound Hongdoushan Formula  Fruiting body of *Ganoderma lucidum* (Leyss. ex. Fr.) Karst., 20g  Fruit of *Psoralea corylifolia* L., 10g  Pseudobulb of *Cremastra appendiculata* (D.Don) Makino, 10g  Rhizome of *Curcuma phaeocaulis* Val., 10g  Bark of *Taxus Chinensis*（Pilger）Rehd., 5g  Skin of *Bufo bufo gargarizans* Cantor, 2g |

Abbreviation: bid, twice a day; N/A, not available; qd, once a day; tid, three times a day.
