# Supplementary material for: Impact of traditional East Asian medicine as an add-on therapy on survival and recurrence after surgery for breast cancer: A systematic review and meta-analysis
Source: Front Pharmacol. 2023 Apr 4;14:1125373. doi: 10.3389/fphar.2023.1125373 (PMC10110841; doi:10.3389/fphar.2023.1125373)
Supplement: Supplementary file 2 [file DataSheet1.docx]

Supplementary Material

Impact of Traditional East Asian Medicine as an Add-on Therapy on Survival and Recurrence after Surgery for Breast Cancer: A Systematic Review and Meta-Analysis

Jee-hyun Yoon, Eun Hye Kim, Su Bin Park, Hayun Jin, Seong Woo Yoon*

*** Correspondence:** Seong Woo Yoon: [stepano212@hanmail.net](mailto:stepano212@hanmail.net)

# Supplementary material 1. Search Strategy

- - 1. Pubmed

#1 “breast neoplasms”[Mesh]

#2 (“breast tumor*” OR “breast cancer” OR “cancer, breast” OR “mammary cancer*”)

#3 (“malignant neoplasm of breast” OR “breast malignant neoplasm*” OR “malignant tumor of breast” OR “breast malignant tumor*”)

#4 (“cancer of breast” OR “cancer of the breast” OR “mammary carcinoma*”)

#5 #1 OR #2 OR #3 OR #4

#6 recurrence[Mesh]

#7 (recurrence* OR recrudescence* OR relapse*)

#8 “neoplasm metastasis”[Mesh]

#9 (“neoplasm metastases” OR metastasis OR metastases OR “metastasis, neoplasm”)

#10 #6 OR #7 OR #8 OR #9

#11 (surger* OR surgical* OR operative* OR operation*)

#12 (postop* OR post-op* OR postoperat* OR post-operat* OR postsurg* OR post-surg*)

#13 #11 OR #12

#14 “drugs, chinese herbal”[MeSH]

#15 “plants, medicinal”[MeSH]

#16 “herbal medicine”[MeSH]

#17 “medicine, kampo”[MeSH]

#18 “medicine, korean traditional”[MeSH]

#19 “medicine, chinese traditional”[MeSH]

#20 (“traditional korean medicine” OR “traditional chinese medicine” OR “traditional oriental medicine” OR “kampo medicine” OR herb* OR decoction* OR botanic*)

#21 #12 OR #13 OR #14 OR #15 OR #16 OR #17 OR #18

#22 #5 AND #10 AND #13 AND #21

- - 1. CNKI

#1 乳腺癌

#2 乳腺肿瘤

#3 乳癌

#4 乳房癌

#5 乳房肿瘤

#6 Breast Cancer

#7 OR / 1-6

#8 术后

#9 Postoperative

#10 OR / 8-9

#11 复发

#12 转移

#13 recurrence

#14 metastasis

#15 OR / 11-14

#16 中药

#17 中医

#18 汤

#19 饮

#20 散

#21 汤剂

#22 丸

#23 中成药

#24 方剂

#25 中西医结合

#26 颗粒

#27 胶囊

#28 口服液

#29 Capsule

#30 Powder

#31 Herbal medicine

#32 Traditional Chinese medicine

#33 TCM

#34 Traditional medicine

#35 Decoction

#36 Chinese medicine

#37 OR / 16-36

#38 #7 AND #10 AND #15 AND #37

- - 1. EMBASE

#1 'breast tumor'/exp

#2 'neoplasm*, breast' OR 'breast tumor*' OR 'tumor*, breast' OR 'breast cancer' OR 'cancer, breast' OR 'mammary cancer*' OR 'cancer*, mammary'

#3 'malignant neoplasm of breast' OR 'breast malignant neoplasm*' OR 'malignant tumor of breast' OR 'breast malignant tumor*'

#4 'cancer of breast' OR 'cancer of the breast' OR 'mammary carcinoma*'

#5 #1 OR #2 OR #3 OR #4

#6 'recurrent disease' /exp

#7 recurrence* OR recrudescence* OR relapse*

#8 'metastasis'/exp

#9 'metastases, neoplasm' OR 'neoplasm metastases' OR metastases OR 'metastasis, neoplasm'

#10 #6 OR #7 OR #8 OR #9

#11 postop* OR 'post op*' OR postoperat* OR 'post operat*' OR postsurg* OR 'post surg*'

#12 surger* OR surgical* OR operative* OR operation*

#13 #11 OR #12

#14 'chinese medicine'/exp

#15 'korean medicine'/exp

#16 'kampo medicine'/exp

#17 #14 OR #15 #16

#18 #5 AND #10 AND #13 AND #17

#19 [controlled clinical trial]/lim OR [randomized controlled trial]/lim

#20 #18 AND #19

- - 1. Cochrane Library

#1 MeSH descriptor: [Breast Neoplasms] explode all trees

#2 “Neoplasm*, Breast” OR “Breast Tumor*” OR “Tumor*, Breast” OR “Breast Cancer” OR “Cancer, Breast” OR “Mammary Cancer*” OR “Cancer*, Mammary”

#3 “Malignant Neoplasm of Breast” OR “Breast Malignant Neoplasm*” OR “Malignant Tumor of Breast” OR “Breast Malignant Tumor*”

#4 “Cancer of Breast” OR “Cancer of the Breast” OR “Mammary Carcinoma*”

#5 #1 OR #2 OR #3 OR #4

#6 MeSH descriptor: [Recurrence] explode all trees

#7 Recurrence* OR Recrudescence* OR Relapse*

#8 MeSH descriptor: [Neoplasm Metastasis] explode all trees

#9 “Metastases, Neoplasm” OR “Neoplasm Metastases” OR Metastasis OR Metastases OR “Metastasis, Neoplasm”

#10 #6 OR #7 OR #8 OR #9

#11 Postop* OR Post-op* OR Postoperat* OR Post-operat* OR Postsurg* OR Post-surg*

#12 Surger* OR Surgical* OR Operative* or Operation*

#13 #11 OR #12

#14 MeSH descriptor: [Medicine, Kampo] explode all trees

#15 MeSH descriptor: [Medicine, Korean Traditional] explode all trees

#16 MeSH descriptor: [Medicine, Chinese Traditional] explode all trees

#17 #14 OR #15 OR #16

#18 #5 AND #10 AND #13 AND #17

5) KMBASE

#1 breast cancer AND recurrence AND Korean medicine

#2 breast cancer AND recurrence AND traditional medicine

#3 breast cancer AND recurrence AND herb

#4 OR / 1-3

6) KISS

#1 breast cancer AND recurrence AND Korean medicine

#2 breast cancer AND recurrence AND traditional medicine

#3 breast cancer AND recurrence AND herb

#4 OR / 1-3

7) KCI

#1 breast cancer AND recurrence AND Korean medicine

#2 breast cancer AND recurrence AND traditional medicine

#3 breast cancer AND recurrence AND herb

#4 OR / 1-3

8) OASIS

#1 breast cancer AND recurrence AND Korean medicine

#2 breast cancer AND recurrence AND traditional medicine

#3 breast cancer AND recurrence AND herb

#4 OR / 1-3

9) CiNii

#1 (breast cancer OR breast neoplasms) AND (recurrence OR metastasis) AND (postop* OR postsurg*) AND (herb OR korean medicine OR chinese medicine OR kampo medicine OR herbal medicine)
